# The Effect of Exogenous Zinc Concentration on the Responsiveness of MC3T3-E1 Pre-Osteoblasts to Surface Microtopography: Part I (Migration)

**DOI:** 10.3390/ma6125517

**Published:** 2013-11-27

**Authors:** Kathryn Dorst, Derek Rammelkamp, Michael Hadjiargyrou, Dilip Gersappe, Yizhi Meng

**Affiliations:** 1 Department of Materials Science and Engineering, Stony Brook University, Stony Brook, NY 11794-2275, USA; E-Mails: Kathryn.Dorst@StonyBrook.edu (K.D.); Derek.Rammelkamp@StonyBrook.edu (D.R.); Dilip.Gersappe@StonyBrook.edu (D.G.); 2 Department of Life Sciences, New York Institute of Technology, Old Westbury, NY 11568-8000, USA; E-Mail: Mhadji@NYIT.edu; 3 Department of Chemical and Molecular Engineering, Stony Brook University, Stony Brook, NY 11794-2275, USA

**Keywords:** zinc, osteoblast, polydimethylsiloxane, micropatterns, contact guidance

## Abstract

Initial cell-surface interactions are guided by the material properties of substrate topography. To examine if these interactions are also modulated by the presence of zinc, we seeded murine pre-osteoblasts (MC3T3-E1, subclone 4) on micropatterned polydimethylsiloxane (PDMS) containing wide (20 µm width, 30 µm pitch, 2 µm height) or narrow (2 µm width, 10 µm pitch, 2 µm height) ridges, with flat PDMS and tissue culture polystyrene (TC) as controls. Zinc concentration was adjusted to mimic deficient (0.23 µM), serum-level (3.6 µM), and zinc-rich (50 µM) conditions. Significant differences were observed in regard to cell morphology, motility, and contact guidance. We found that cells exhibited distinct anisotropic migration on the wide PDMS patterns under either zinc-deprived (0.23 µM) or serum-level zinc conditions (3.6 µM). However, this effect was absent in a zinc-rich environment (50 µM). These results suggest that the contact guidance of pre-osteoblasts may be partly influenced by trace metals in the microenvironment of the extracellular matrix.

## 1. Introduction

Alterations in physical and chemical characteristics of biomaterial surfaces can modulate cellular behavior via complex events in the extracellular matrix (ECM). In particular, variations in microtopography, mechanical properties (Young’s modulus), and surface chemistry can promote cell attachment and migration [1,2,3,4]. The initial formation and development of the ECM can determine cell fate as it undergoes remodeling that is required for tissue homeostasis.

Bone development and regeneration are dynamic processes that are intricately regulated by the interplay between cells and their ECM [5]. These processes are critical in driving osteogenic differentiation by recruiting osteoblast precursor cells from the surrounding tissues [6,7,8,9]. Recently, it was shown that the motility of mesenchymal cells was enhanced as soon as one day after treatment with osteogenic medium through the increased activation of the small GTPases, Cdc24 and Rac1, which are critical for mesenchymal cell migration [10]. As such, directional cell migration is crucial to the successful recruitment of these precursor cells, however, its precise role in the regulation of the differentiation of osteoprogenitor cells has not been fully established.

Engineered biomimetic anisotropic topographies (e.g., microscale ridges/grooves) have been shown to promote the adhesion of human osteoblasts [11,12,13], osteosarcoma cells [1,14], murine osteoblast-like and mesenchymal stem cells [15,16]. These microfabricated patterns can be designed to induce cell elongation and alignment, resulting in an anisotropic rearrangement of the cell, reorganization of its cytoskeleton, and the re-distribution of the focal adhesions [17,18]. In contrast, the cytoskeleton of cells adhered on a planar surface is randomly structured [19] and they migrate in a random walk fashion [19]. When anisotropy is introduced into the system, such as via a mechanical or chemical gradient, cells deviate from this random walk behavior and exhibit directional migration. Contact guidance, which is bi-directional locomotion along an axis of anisotropy, is one specific example of biased cell migration [20]. Cells that exhibit a directional bias in their motion alter the conformation of their cell body and the distribution of adhesion complexes, which can result in the initiation of signaling events involved in actin polymerization as well as the formation of new focal contacts at the leading edge [21].

Surface roughness can also modulate cellular response to nutritional factors, such as vitamin D, as shown in an *in vitro* osteoblast model [22]. Because trace metals are essential for bone health [23], we hypothesized that they may also affect the responsivity of osteoblasts to microtopography. Recently, the homeostatic regulation of zinc has been implicated in impaired skeletal growth [24,25,26], and zinc deficiency was shown to impede synthesis of bone matrix proteins and calcification of the ECM [26,27,28]. A secondary messenger involved in many signaling pathways [24,27,29,30,31,32], zinc plays a critical role in cell survival and migration [24,31,32,33]. Zinc homeostasis associated with bone growth is facilitated by transporter proteins from the Slc39/Zip and Slc30/ZnT families [34,35,36]. Of particular importance are Zip13 and Zip14, which are involved in intracellular zinc distribution and regulation of mammalian systemic growth via the BMP/TGF-β and G-protein coupled receptor (GPCR)-mediated signaling pathways [34,35], as well as Znt5, which is critical for osteoblast maturation *in vitro* and maintenance of bone density *in vivo* [36].Recent studies have shown that zinc is involved in the late-stage expression of RUNX2, a gene that has been suggested to be essential for recruiting migratory osteoblasts during bone mineralization [37,38]. Therefore early stage migration could be closely linked to ECM turnover and bone mineral production, and could be a useful metric for predicting success of bone formation.

In order to determine the role of exogenous zinc on the migratory behavior of osteoblasts, we cultured MC3T3-E1 cells on micropatterned substrates under zinc-deficient (0.23 µM), serum-level (3.6 µM), and zinc-rich (50 µM) conditions. We hypothesized that early stage (within the first 24 h) interactions at the cell-material interface are interdependent on both substrate microtopography and zinc serum concentration.

## 2. Results and Discussion

### 2.1. Results

#### 2.1.1. Cell Coverage

**Figure 1:**
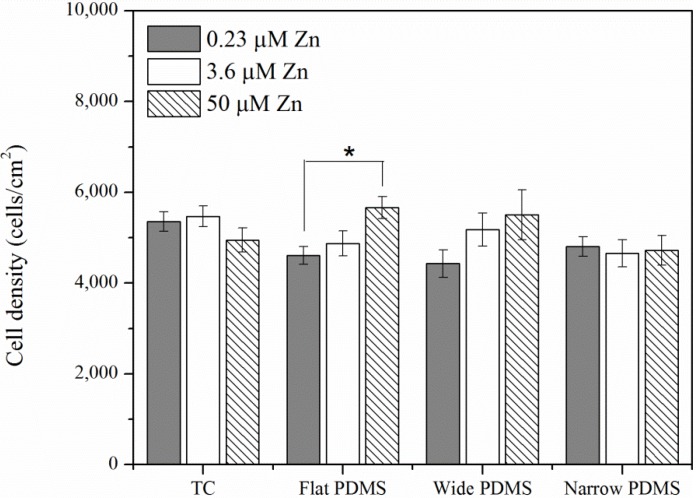
Area density of MC3T3-E1 (subclone 4) pre-osteoblast cells on various substrates with modifications of zinc concentrations after incubation for 24 h. A single asterisk (*) indicates a *p* < 0.01 level of significance.

At twenty-four hours post-plating, area coverage of MC3T3-E1 pre-osteoblasts in general was similar on both tissue culture polystyrene (TC) and polydimethylsiloxane (PDMS) (Figure 1). The only statistically significant increase in cell density was observed when cells were cultured on the flat PDMS at the highest zinc concentration (50 µM). A similar effect, although not statistically significant (*p* = 0.5), was also observed for cells on wide PDMS patterns (Figure 1). In contrast, cell coverage on the other substrates was not affected by exogenous zinc levels.

#### 2.1.2. Actin Analysis and Morphology

**Figure 2:**
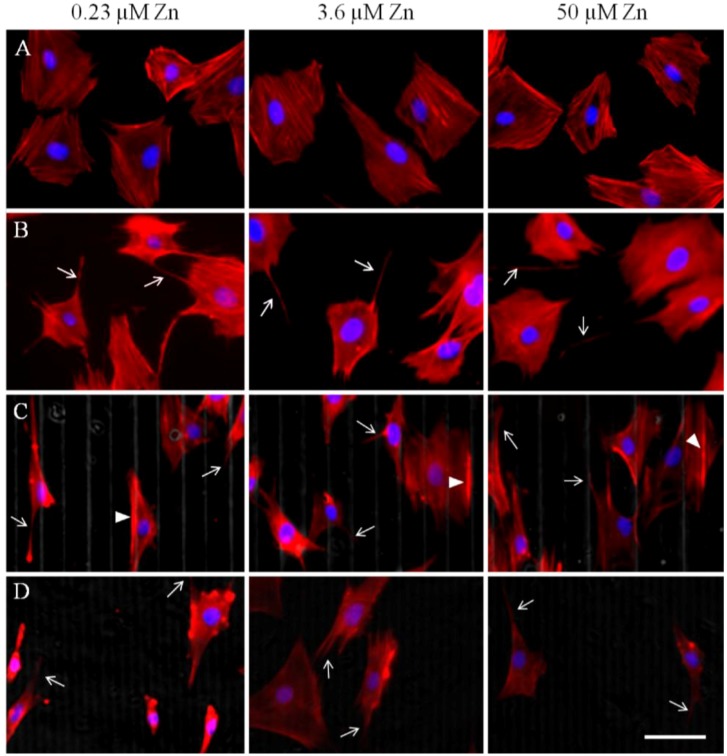
Immunofluorescence micrographs of F-Actin (visualized in the red channel) 24 h after seeding at various zinc concentrations on (**A**) tissue culture polystyrene (TC); (**B**) flat polydimethylsiloxane (PDMS); (**C**) wide PDMS pattern; and (**D**) narrow PDMS pattern. Nuclei are visualized in the blue channel. Arrows indicate discrete lamellipodia protrusions while arrowheads in (C) indicate actin clustering on the edge of a micropattern. Bar = 50 µm.

**Figure 3:**
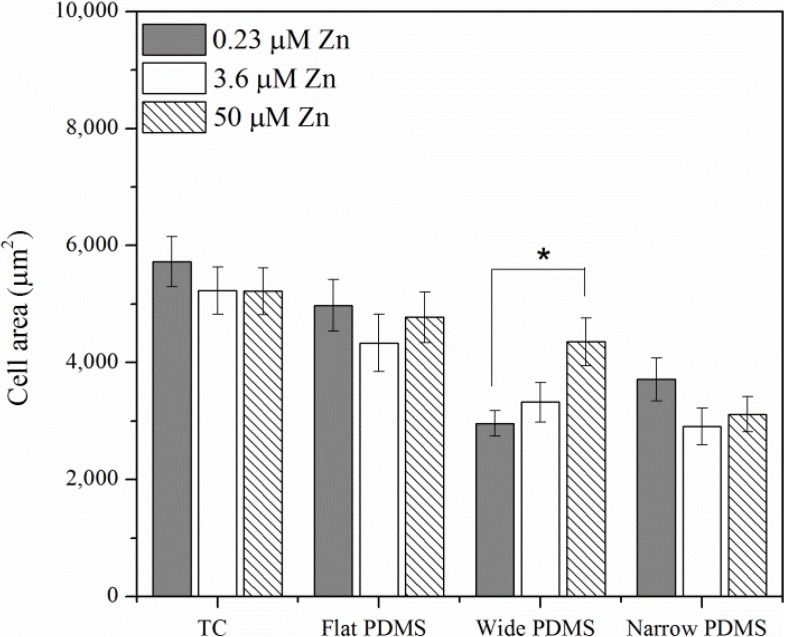
Cell spreading area computed from fluorescence micrographs in Figure 2A–D. A single asterisk (*) indicates a *p* < 0.01 level of significance.

Twenty-four hours after seeding, cells had sufficiently adhered to their substrates and F-actin stress fibers were clearly visible on all substrates, as expected (Figure 2A–D). Cell spreading on all the substrates was significantly different: cells on TC and flat PDMS appear to be more spread out and thus larger in area (Figure 2A,B and Figure 3) compared to those on the patterned substrates (Figure 2C,D and Figure 3). Additionally, cells on PDMS surfaces appeared to have more protruding lamellipodia than on TC, typically at the trailing edge (arrows in Figure 2B–D). Analysis of the cell spreading area suggests that zinc concentration only had a significant effect on those cells that were cultured on the wide PDMS pattern, which appeared to be better adhered with increasing zinc (Figure 3).

**Figure 4:**
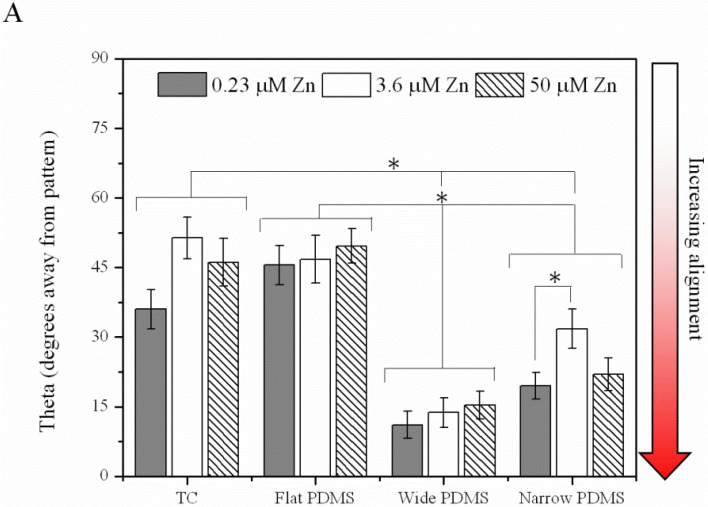

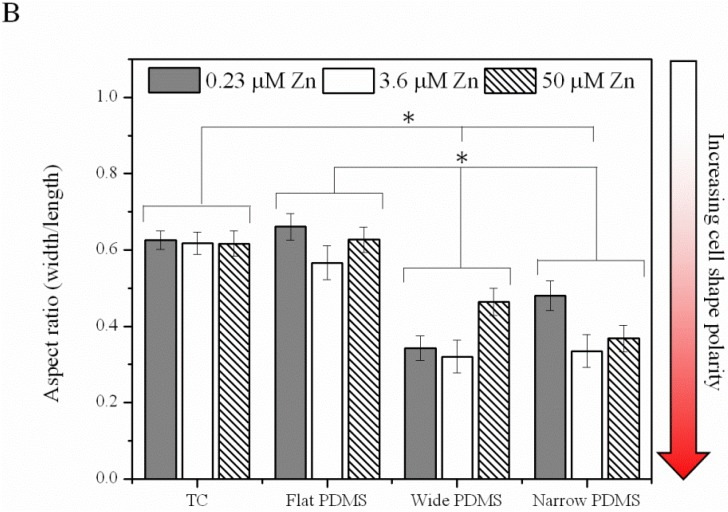
(**A**) Cell alignment computed from Figure 2A–D, where 0 degrees indicates complete alignment with patterned ridges/*y*-axis. (**B**) Cell shape aspect ratio was calculated from the same set of images and represents the use of ImageJ’s ellipse fitting tool to determine ratio of cell width/length. A single asterisk (*) indicates a *p* < 0.01 level of significance.

The orientation of the actin cytoskeleton was strongly correlated with the type of substrate and topography (Figure 4A). On the wide PDMS micropatterns (20/30 µm), the actin fibers were most clearly aligned (low theta values) with respect to the longitudinal direction of the pattern (Figure 2C and Figure 4A), whereas those on the narrow PDMS micropatterns (2/10 µm) were not as aligned (higher theta values), as several cells were observed to be oriented diagonally (Figure 2D and Figure 4A). Additionally, actin seemed to cluster on the edges of the wide pattern (arrowheads in Figure 2C). On TC and flat PDMS, the actin fibers were not aligned in any particular orientation, as would be expected for a non-patterned surface (Figure 2A,B and Figure 4A). Cells on both TC and flat PDMS were also elongated to a similar extent (Figure 4B), but with no statistical differences between substrate type or zinc level.

#### 2.1.3. Cell Migration

**Figure 5:**
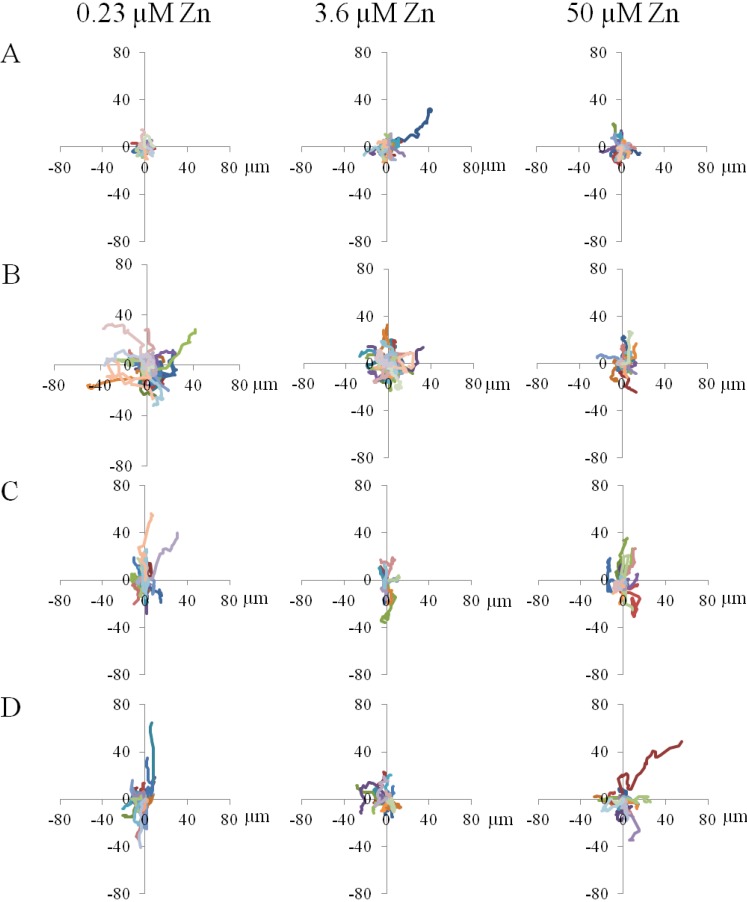

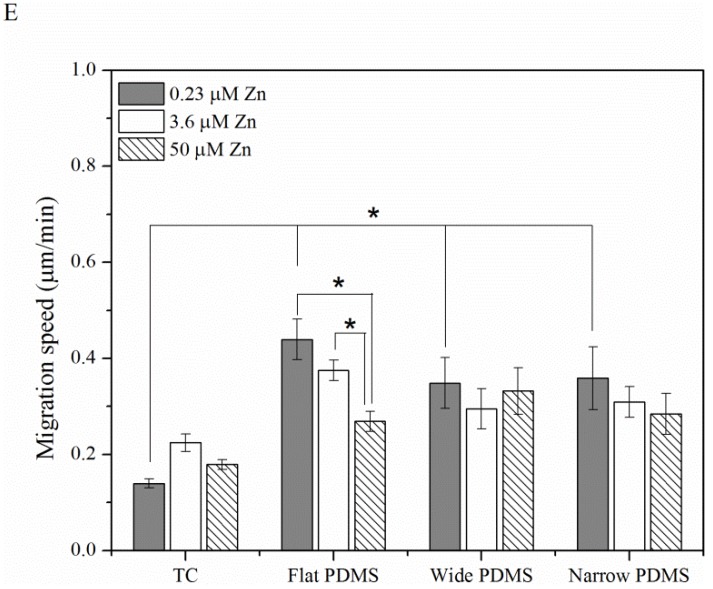
Displacement paths of subclone 4 MC3T3-E1 cells in various zinc-modified media after attaching for 2 hours on (**A**) TC; (**B**) flat PDMS; (**C**) 20 µm wide/30 µm wide patterned PDMS ridges; and (**D**) 2 µm wide/10 µm narrow patterned PDMS ridges. Colored lines represent the migration paths tracked using time-lapse video microscopy; (**E**) Migration speeds of MC3T3-E1 cells on various substrates with modified zinc levels. A single asterisk (*) indicates a *p* < 0.01 level of significance.

Average migration speed, displacement, and directionality of cell movement varied significantly with substrate type. Specifically, cells on TC typically did not travel far from their starting point during the entire two hours of time-lapse video capture (Figure 5A; Supplementary Videos 1–3) and migration was randomly directed. Cell migration on flat PDMS (Figure 5B; Supplementary Videos 4–6) also took a random pattern, while cells on the wide PDMS patterns traveled much further, in line with the pattern (on top of the ridges; Figure 5C; Supplementary Videos 7–9). On the narrow PDMS patterns cell migration appeared more random than on the wide patterns (Figure 5D; Supplementary Videos 10–12).

In general, cell migration was noticeably slowest on TC (0.14–0.22 µm/min) and was not found to be dependent on zinc (Figure 5E). In contrast, cell migration on flat PDMS was considerably faster (0.27–0.44 µm/min) and showed a steady decrease with increasing zinc concentration (Figure 5E). The migratory behavior of MC3T3-E1 cells on flat PDMS is similar to previous reports on breast cancer cells, where the addition of exogenous zinc also attenuated migration [39,40]. Motility of cells on both wide (0.29–0.34 µm/min) and narrow (0.28–0.36 µm/min) PDMS patterns was similar to that on flat PDMS, however zinc concentration did not have a significant effect (Figure 5E; *p* = 0.35–0.86). Under zinc-deprived (0.23 µM) and serum-level zinc (3.6 µM) conditions, migration on patterned PDMS was reduced compared to flat PDMS, but this reduction was also not significant (*p* = 0.11–0.74). Under zinc-rich conditions (50 µM), migration speed did not vary much at all among the three PDMS surfaces. This suggests that cells are less responsive to substrate microtopography when zinc is abundant.

#### 2.1.4. Contact Guidance

**Figure 6:**
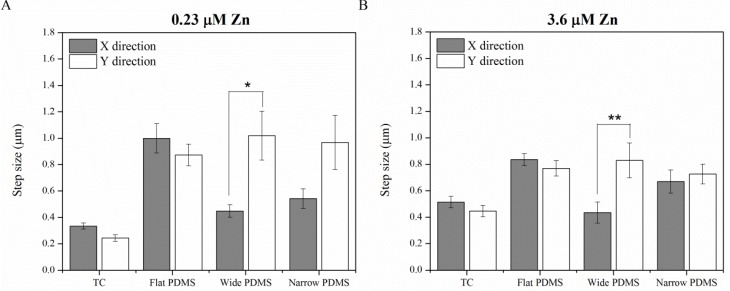

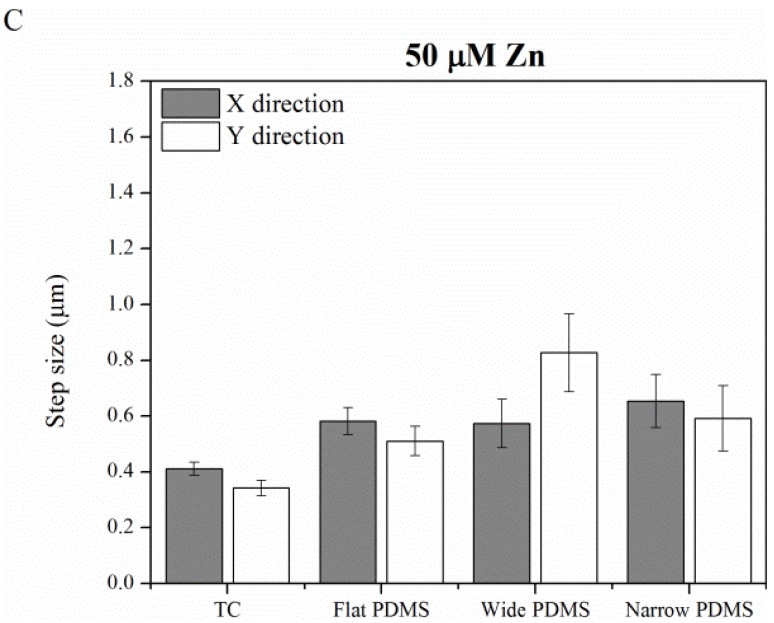
Average cell step size in *x* and *y* directions with (**A**) zinc-deprived media; (**B**) serum-level zinc media; and (**C**) zinc-rich media. A single asterisk (*) indicates a *p* < 0.01 difference while double asterisks (**) indicate a *p* < 0.05 difference.

At all concentrations of zinc, noticeable differences in cell step size, as well as distinction between step direction in *x* and *y*, were seen (Figure 6). At 0.23 µM zinc (zinc-deprived) cells on wide patterned PDMS demonstrated a statistically significant preference for migrating in the *y* direction, along the lengths of the PDMS ridges (Figure 6A). Cells on narrow patterned PDMS also exhibited a slight preference for migrating along the ridges, but not at a significant level (*p* = 0.064). For cells on TC and flat PDMS there was no preference for either direction. At 3.6 µM zinc (serum-level) only cells on the wide PDMS pattern retained their predilection for migration along the ridges, whereas cells on the narrow PDMS pattern behaved as if they were on flat PDMS (Figure 6B). At even higher zinc concentration (50 µM), step size was not significantly different in *x* or *y* direction for cells on all surfaces (Figure 6C).

### 2.2. Discussion

Motility of anchorage-dependent cells is known to be influenced by substrate material properties such as Young’s modulus, surface energy, and microtopography. However, the interplay between trace metals and cell migration is not as well understood. In our study, adhesion, morphology and migratory behavior were examined for MC3T3-E1 pre-osteoblasts cultured in various concentrations of exogenous zinc on a microfabricated anisotropic elastomer (PDMS), which has been frequently used to demonstrate contact guidance in cells with a motile phenotype [18,41]. Non-patterned PDMS was also used, as well as standard tissue culture polystyrene as controls.

Cell coverage within 24 h of seeding was not distinctly different on the four types of surfaces, indicating that neither substrate nor zinc concentration played a significant role in early stage cell survival. The morphology of the MC3T3-E1 cells was round on both of the flat substrates (TC, non-patterned PDMS). In the case of motility, cells on TC did not exhibit a dose dependence on zinc, while migration of the MC3T3-E1 cells on flat PDMS was very sensitive to zinc concentration, similar to previous studies on cancer cell migration [39,40,42]. This suggests that the mode of migration on PDMS is fundamentally different than on TC for the pre-osteoblasts. In contrast, cells cultured on the patterned PDMS surfaces exhibited an elongated spindle-like morphology, typical of mesenchymal cells [7], and their migration speed was not significantly altered by zinc concentration. The spindle-like morphology has been reported for cells cultured on softer substrates [43], and therefore we believe that the compliance of the PDMS is partially responsible for the elongation of the MC3T3-E1 cells and their ability to migrate faster than cells on TC. While tracking cell movements, we also observed many more lamellipodia in cells cultured on PDMS compared to TC, suggesting that the elastomeric substrate is more conducive to the formation of cell protrusions than polystyrene, which could explain the increase in migration speed. In addition, the cells traveling on the larger patterned surfaces had increased numbers of small lamellipodial protrusions, particularly when interacting with the edges of the PDMS ridges. This indicates a more motile phenotype, which is in agreement with the higher migration speeds we observed.

In addition to cell polarity, we observed a significant increase in actin alignment of the MC3T3-E1 cells on patterned PDMS, similar to previous studies of human osteosarcoma cells on PDMS [1,14] as well as murine osteoblast-like and mesenchymal stem cells on micropatterned titanium and silicon [15,16]. The elongation and alignment of the cells results from an anisotropic rearrangement of the cytoskeleton, which re-distributes focal adhesions [17,18]. In our study, the stress fibers of MC3T3-E1 cells on both TC and flat PDMS were randomly oriented, which was expected based on previous studies on flat substrates [19]. There was, however, a visible increase in actin aggregation in the MC3T3-E1 cells along the edges of the wide PDMS ridges, but this clustering was not as pronounced in cells cultured on the narrow PDMS patterns.

Shifts in the spatial distribution of focal adhesions are responsible for regulating cell-substrate adhesion strength [44] and are dependent on both the adhesivity of the ECM and the mechanical compliance of the substrate [45]. A re-distribution of these adhesion complexes would alter the magnitude of cell traction forces [43], and in turn, would influence cell motility. Previous studies using a rat macrophage model [41] have suggested that ligand clustering on anisotropic surfaces is associated with increased substrate adhesiveness and is responsible for the contact guidance phenomenon [21]. The migration trajectories of the MC3T3-E1 cells, especially those cultured on wide (20 µm) PDMS micropatterns, exhibited some extent of biased migration along the length of the PDMS micropattern, particularly at the lower zinc concentrations (Supplementary Videos 7 and 8). Step size analysis further confirmed that the wide PDMS micropattern was associated with anisotropic migration, as cells distinctly preferred to migrate parallel to the ridges in the *y*-direction. On the other hand, migration in the *y*-direction for cells on the narrow PDMS micropatterns under zinc-deficient conditions was only slightly significantly different (*p* = 0.064) from that in the *x*-direction, indicating that the requirements for contact guidance vary with surface microtopography.

Taken together, these results suggest that the extent to which exogenous zinc levels alter pre-osteoblast motility and directional migration strongly depends on surface dimension. While cell motility on flat PDMS distinctly showed dose dependence on zinc concentration, migration speeds on either patterned PDMS or TC showed no clear trend. Our data also suggest that the anisotropic structure of the PDMS surface was responsible for the directional migration observed in MC3T3-E1 cells, however the contact guidance behavior could be altered under zinc-rich conditions. Because zinc is known to play a role in regulating the binding association between collagen and fibronectin [46], it is possible that high levels of zinc directly interfered with contact guidance. Extracellular stimuli can trigger zinc-mediated intracellular signaling events either via ZIP transporters or GPCR receptors such as GPR39, which is involved in migration and metabolic activity [24,47]. Further elucidation of the effects of exogenous zinc on the migratory behavior of pre-osteoblasts can enhance our understanding of bone differentiation and mineralization processes, and improve the design of medical devices for enhanced osseointegration.

## 3. Experimental Section

### 3.1. Microfabrication

Using a negative photoresist (SU-8, Microchem, Newton, MA, USA), we fabricated master molds on a silicon substrate at the Center for Functional Nanomaterials (CFN), Brookhaven National Laboratory (BNL, Upton, NY, USA). Silicon wafers (75 mm diameter, single-polished) with <100> orientation were cleaned with acetone and 2-propanol (3,000 rpm for 30 s) before a 2 µm layer of negative SU-8-2 photoresist (Rohm and Haas; Midland, MI, USA) was deposited by spinning at 2,000 rpm for 30 s. After prebaking for 1 min at 65 °C, micropatterns were transferred from a chrome mask to the resist using a 6 s UV exposure on the Karl Suss MJB3 Mask Aligner (277 Watts). The exposed photoresist was post-baked for 1 min at 95 °C, and the pattern was developed by immersing in propylene glycol monomethyl ether acetate (PGMEA) (Rohm and Haas) for 5–8 min and rinsing with DI water, leaving behind raised SU-8 sections.

Polydimethylsiloxane (PDMS) replicas containing two patterns (20 µm wide with a 30 µm pitch and 2 µm height, and 2 µm wide with a 10 µm pitch and 2 µm height) were prepared as follows: Sylgard 184 (Dow Corning, Midland, MI, USA) prepolymer was mixed with curing agent at a mass ratio of 10:1, and degassed for 20 min. The unpolymerized PDMS was then poured on top of the patterned SU-8 negative master and cured at 65 °C for 1 h. After curing, the positive PDMS patterns were slowly released and plasma etched with 98% oxygen plasma in CFN’s Trion Phantom III Reactive Ion Etcher (Clearwater, FL, USA) in order to create silanol groups at the surface of the polymer, rendering it hydrophilic [48]. Deionized water was added immediately after etching to prevent hydrophobic recovery.

### 3.2. Substrate Preparation

Studies utilized 24-well tissue culture (TC) plates (BD Falcon, Bedford, MA, USA) for cell culture. PDMS samples were sterilized by immersion in 70% ethanol for 2 h in their corresponding wells in the 24-well plate. They were then rinsed twice with Dulbecco’s Phosphate Buffered Saline 1× (PBS; Gibco/Invitrogen) before any further substrate preparation. PDMS samples were functionalized with fibronectin, as a 10 µg/mL solution (Sigma Aldrich, St. Louis, MO, USA) in PBS covered the substrates for 30 min at room temperature. After subsequent rinsing with PBS, bovine serum albumin (BSA; EMD Chemicals; Gibbstown, NJ, USA) was used to block nonspecific protein binding.

### 3.3. Cell Culture and Development

Strongly mineralizing MC3T3-E1 pre-osteoblast cells (subclone 4; American Type Culture Collection (ATCC), Manassas, VA, USA) were maintained in 10 cm TC plates (BD Falcon; Franklin Lakes, NJ, USA) using MEM-α (Gibco/Invitrogen, Grand Island, NY, USA) supplemented with 10% fetal bovine serum (FBS; Hyclone/Thermo Fisher Scientific; Logan, UT, USA) and 1% penicillin-streptomycin (Gibco/Invitrogen). Cells were kept in an incubator at 37 °C with 5% CO_2_, 95% relative humidity.

### 3.4. Preparation of Zinc-Deficient and Zinc-Rich Medium

Zinc-deficient medium was prepared by removing zinc from the FBS used in the alpha MEM, similar to the procedure published by Prasad *et al*. [49] and Cho *et al*. [50]. To prepare zinc-rich medium, the final concentration of zinc was adjusted to 50 µM with zinc sulfate.

### 3.5. Cell Coverage

Cells were seeded at a density of 5,000 cells/cm^2^ in a 24-well TC plate and incubated (5% CO_2_, 95% humidified) at 37 °C for 24 h. Samples were rinsed with PBS and fixed using 3.7% formaldehyde (J.T. Baker/Mallinckrodt Baker Inc.; Phillipsburg, NJ, USA) for 15 min. The samples were then rinsed with PBS and stained using 2.5 µg/mL of 4′,6-Diamidino-2-Phenylindole Dihydrochloride (DAPI; Sigma, St. Louis, MO, USA) in PBS for 5 min covered at room temperature and then rinsed twice with PBS. Approximately 150 fluorescent images were captured for each sample. Images were quantified using ImageJ software and analysis was performed to calculate the average cell density per sample.

### 3.6. Actin Analysis

F-actin distribution was visualized with a standard inverted fluorescence microscope (IX51; Olympus Instruments, Melville, NY, USA). Cells seeded at 5,000 cells/cm^2^ were maintained at 37 °C for 24 h to allow complete attachment to the substrate. After fixing with 3.7% formaldehyde, the cells were permeabilized with 0.4% Triton X-100 for 7–8 min. The samples were then rinsed with PBS and subsequently stained with 1:100 Alexa Fluor 594 phalloidin (Invitrogen, Grand Island, NY, USA). Using ImageJ, the average spreading area was calculated by converting the number of pixels^2^ in the cell area into micrometers^2^.

The ImageJ particle measurement plugin was used to fit each cell area to an ellipse shape that represented the ratio of width/length of the cell to determine elongation and alignment to the *y*-axis/patterns. Actin alignment was defined as the angle formed between the major axis of the cell and the direction of the micropattern (*y*-axis), such that a complete, parallel alignment was indicated by 0°. For non-patterned samples, the *y*-axis was chosen arbitrarily to compare amongst samples. Around 50 cells from three independent experiments were analyzed per sample.

### 3.7. Cell Migration: Time-Lapse Microscopy

To capture the migration behavior of cells on the optically transparent substrates, cells were first seeded at 10,000 cells/cm^2^ and incubated at 37 °C for 2 h to allow complete attachment. The medium was then replaced with CO_2_-independent medium (supplemented with 10% FBS, 1% penicillin/streptomycin, and 1% L-glutamine, plus zinc additions) and cells were maintained on a 37 °C heated stage. Phase contrast micrographs were captured at 3 min intervals with a CCD camera attached to the microscope, for a total duration of 2 h. ImageJ software was used to calculate migration speeds and displacement using a manual tracking plugin.

### 3.8. Numerical Analysis of Migration

To analyze the directional dependent motility of MC3T3-E1 cells, a Matlab algorithm was used to calculate step length as follows (Equations (1) and (2)) [51]:

(1) $${\mathrm{SL}}_{x}=\;\frac{\left|x_{2}-x_{1}\right|+\left|x_{3}-x_{2}\right|+\text{…}+\left|x_{n}-x_{n-1}\right|}{n-1}$$

(2) $${\mathrm{SL}}_{y}=\;\frac{\left|y_{2}-y_{1}\right|+\left|y_{3}-y_{2}\right|+\text{…}+\left|y_{n}-y_{n-1}\right|}{n-1}$$

where SL*_x_* and SL*_y_* are defined as the step size in the *x*- and *y*-direction, respectively.

### 3.9. Statistics

Each experiment consisted of a minimum of three replicates per treatment group. Statistical analysis was performed using an unpaired student *t*-test to and differences were considered significant at the level of *p* < 0.01.

## 4. Conclusions

Using an *in vitro* model, we demonstrated that modifying the level of exogenous zinc can alter the extent to which pre-osteoblasts respond to surface microtopography. Specifically, contact guidance was exhibited on 20 µm wide PDMS patterns under either zinc-deprived (0.23 µM) or serum-level zinc conditions (3.6 µM), but was absent in a zinc-rich environment (50 µM). These results suggest that trace metals in the microenvironment of the extracellular matrix can interfere with the migratory behavior of pre-osteoblasts, which may have implications in downstream events such as differentiation and mineralization.

## Supplementary Materials

Supplementary File 1

Supplementary File 2

Supplementary File 2

Supplementary File 2

Supplementary File 2

Supplementary File 2

Supplementary File 2

Supplementary File 2

Supplementary File 2

Supplementary File 2

Supplementary File 2

Supplementary File 2
